# mHealth Supportive Care Intervention for Parents of Children With Acute Lymphoblastic Leukemia: Quasi-Experimental Pre- and Postdesign Study

**DOI:** 10.2196/mhealth.9981

**Published:** 2018-11-19

**Authors:** Jingting Wang, Doris Howell, Nanping Shen, Zhaohui Geng, Fulei Wu, Min Shen, Xiaoyan Zhang, Anwei Xie, Lin Wang, Changrong Yuan

**Affiliations:** 1 School of Nursing Second Military Medical University Shanghai China; 2 Department of Supportive Care Princess Margaret Cancer Center Toronto, ON Canada; 3 Faculty of Nursing University of Toronto Toronto, ON Canada; 4 Department of Nursing Shanghai Children’s Medical Center Shanghai China; 5 Department of Hematology Children’s Hospital of Soochow University Suzhou China; 6 Department of Hematology and Oncology Shanghai Children’s Medical Center Shanghai China; 7 Department of Infectious Disease Children’s Hospital of Soochow University Suzhou China; 8 United States Census Bureau Washington, DC United States; 9 School of Nursing Fudan University Shanghai China

**Keywords:** acute lymphoblastic leukemia, effectiveness, mHealth, mobile phone, parent, supportive care

## Abstract

**Background:**

Acute lymphoblastic leukemia (ALL) is the most common childhood malignancy. Caring for children with ALL is challenging for parents. A mobile health (mHealth) supportive care intervention was developed to meet parents’ needs.

**Objective:**

This study aims to evaluate the potential effectiveness of this mHealth supportive care intervention on emotional distress, social support, care burden, uncertainty in illness, quality of life, and knowledge.

**Methods:**

We conducted a quasi-experimental pre- and postdesign study from June 2015 to January 2016. In total, 101 parents were enrolled in the study, with 50 in the observation group and 51 in the intervention group. Parents in the observation group received the standard health education and were observed for 3 months. Parents in the intervention group received the mHealth supportive care intervention, in addition to the standard health education. The intervention consisted of 2 parts—an Android smartphone app “Care Assistant (CA)” and a WeChat Official Account. The CA with 8 modules (Personal Information, Treatment Tracking, Family Care, Financial and Social Assistance, Knowledge Center, Self- Assessment Questionnaires, Interactive Platform, and Reminders) was the main intervention tool, whereas the WeChat Official Account was supplementary to update information and realize interaction between parents and health care providers. Data of parents’ social support, anxiety, depression, care burden, uncertainty in illness, quality of life, their existing knowledge of ALL and care, and knowledge need were collected before and after the 3-month study period in both groups. For the intervention group, parents’ experience of receiving the intervention was also collected through individual interviews.

**Results:**

Overall, 43 parents in the observation group and 49 in the intervention group completed the study. Results found that the intervention reduced parents’ anxiety (D_int(Post-Pre)_=−7.0 [SD 13.1], D_obs(Post-Pre)_=−0.4 [SD 15.8], t_90_=−2.200, *P*=.03) and uncertainty in illness (D_int(Post-Pre)_=−25.0 [SD 8.2], D_obs(Post-Pre)_=−19.8 [SD 10.1], t_90_=−2.761, *P*=.01), improved parents’ social function (D_int(Post-Pre)_=9.0 [SD 32.8], D_obs(Post-Pre)_=−7.5 [SD 30.3], t_90_=2.494, *P*=.01), increased parents’ knowledge of ALL and care (D_int(Post-Pre)_=28.4 [SD 12.4], D_obs(Post-Pre)_=17.2 [SD 11.9], t90=4.407, *P*<.001), and decreased their need for knowledge (D_int(Post-Pre)_=−9.9 [SD 11.6], D_obs(Post-Pre)_=−1.9 [SD 6.4], t_90_=−4.112, *P*<.001). Qualitative results showed that parents were satisfied with the intervention and their role in the caregiving process.

**Conclusions:**

The mHealth intervention in supporting parents of children with ALL is effective. This study is informative for other future studies on providing mHealth supportive care for parents of children with cancer.

## Introduction

Cancer is the second leading cause of pediatric death in developed countries [1]. Acute lymphoblastic leukemia (ALL) accounts for 26.8% of all pediatric cancer cases and is the most common childhood malignancy among children younger than 15 years [2]. The peak incidence of childhood ALL occurs in children aged 2-4 years [3]. The 5-year survival rate in childhood ALL is around 85% in developed countries [3] and slightly lower at 80% in China [4]. Regardless of a relatively good prognosis, the diagnosis of childhood ALL is still a life-altering event for parents. Parents are often challenged with taking on the tasks of caring for their children with symptom and emotion management when inadequately equipped with knowledge of the disease and caregiving. In the meantime, parents themselves are in need of supportive care in health, emotion, finance, and social support.

Supportive care was first described in 1994 as “the provision of necessary services as defined by those living with or affected by cancer to meet their physical, psychosocial, informational and spiritual needs during the prediagnostic, diagnostic, treatment, and follow-up phases, while encompassing issues of survivorship, palliation, and bereavement” [5]. In 2000, Cancer Care Ontario in Canada developed the Supportive Care Needs Framework and outlined 7 domains of need, including practical, spiritual, social, psychological, informational, emotional, and physical needs [6]. The Supportive Care Needs Framework was developed to help guide health professionals to meet the supportive care needs of patients with cancer along the disease continuum [6]. A recent study found that parents of children with cancer have the needs for these aspects of supportive care—communication with physician, timely provision of information, and suitable and accessible psychosocial care [7].

Traditionally, support interventions for parents were delivered through face-to-face education and telephone follow-up. Two studies on traditional supportive care for Chinese parents showed effects in reducing parents’ emotional distress and in improving parents’ knowledge of care, satisfaction with care, and relationship with health care providers [8,9].

**Figure 1:**
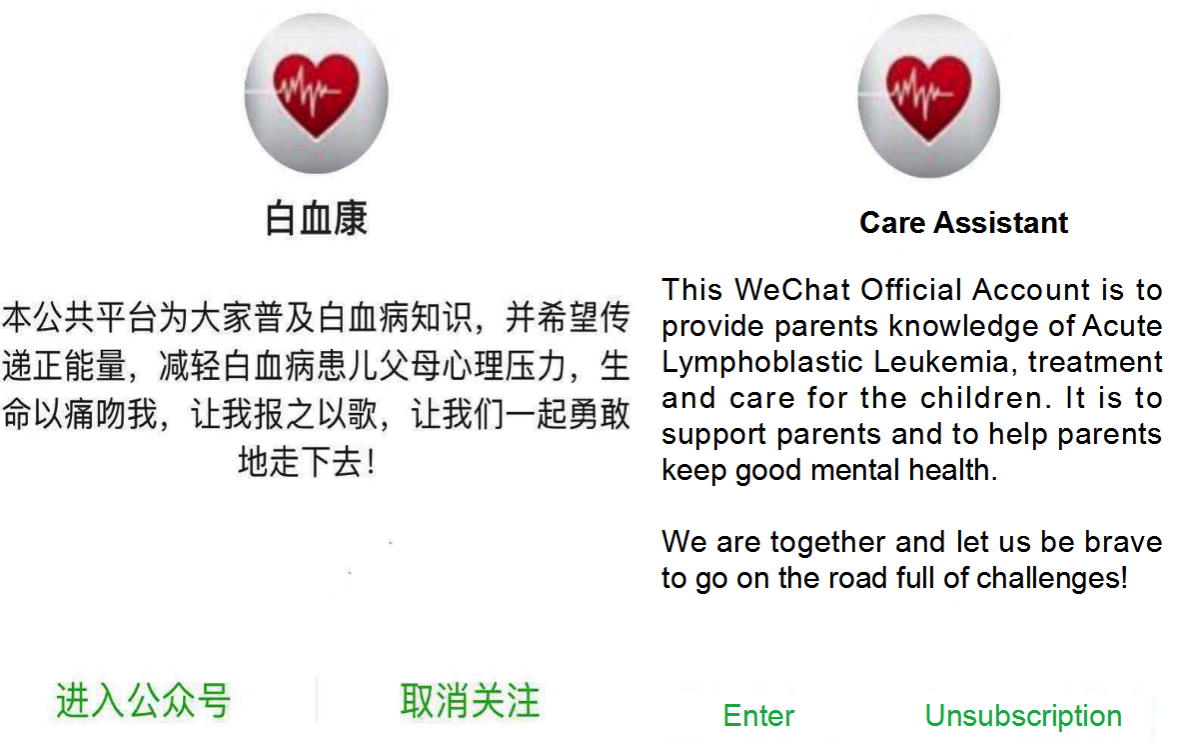
WeChat Official Account welcome page.

In addition, systematic reviews indicated that supportive care interventions improved cancer caregivers’ psychological status, emotion, well-being, disease-related knowledge, quality of life, and coping strategy [10]. However, the traditional modes of delivering interventions have a major disadvantage of poor accessibility. For example, some parents may be less motivated to attend intervention training courses or unable to find substitute caregiver for their children to allow them to attend the training [10].

Mobile health (mHealth) is an emerging technology to make supportive care more accessible for parents of children with ALL. mHealth interventions can be used to manage side effects, improve drug adherence, provide cancer care information, plan and follow-up care, and detect and diagnose cancer [11,12]. Smartphone apps are important mHealth tools and tend to be easy to be acquired. Smartphone apps allow patients to access health information and health care services anytime and anywhere [12]. Studies have documented the feasibility and effectiveness of mobile apps in supporting adult patients with cancer and survivors in health care and clinical practice [13-16]. However, to the best of our knowledge, there has not been much use of apps in supporting parents of pediatric patients with cancer.

**Figure 2:**
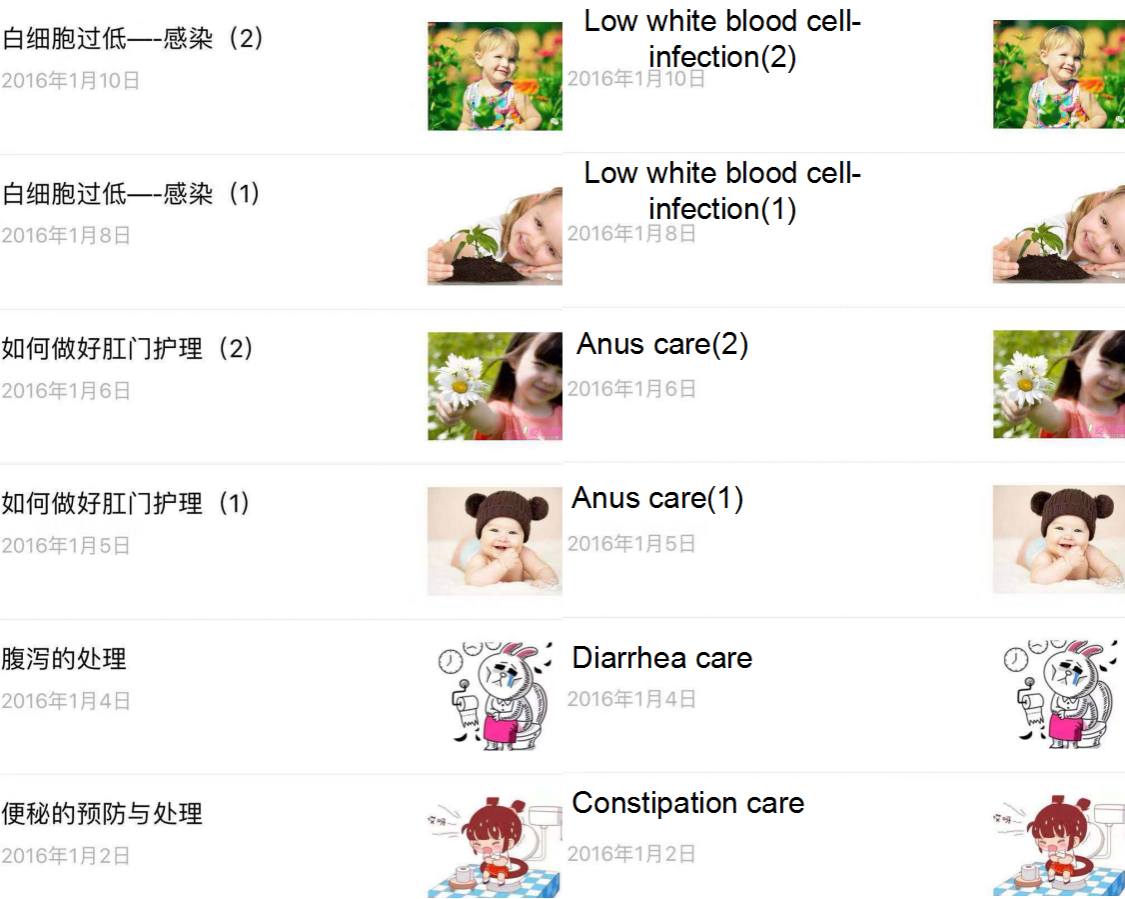
WeChat Official Account health education article example.

To meet the supportive care needs of parents of children with ALL, we designed and implemented a mHealth supportive care intervention that consisted of 2 parts—a smartphone app called “Care Assistant (CA)” that runs on the Android system with good usability [17,18] and a WeChat Official Account. WeChat is a multipurpose social media mobile app that is the most popular in China. The WeChat Official Account is a module of WeChat, which can publish papers to users who subscribe to it. In this study, the CA was the main intervention tool, whereas WeChat was supplementary. The CA has 8 modules as follows: Personal Information, Treatment Tracking, Family Care, Financial and Social Assistance, Knowledge Center, Self-Assessment Questionnaires, Interactive Platform, and Reminders [17]. The selected screenshots to visualize the app can be found in former published papers [17,18]. The WeChat Official Account was added to this intervention because of parents’ needs for information across the children’s illness trajectory. It is a cost-saving strategy to update information and realize interaction between parents through the addition of a WeChat Official Account. Figures 1 and 2 are selected screenshots provided to visualize the WeChat Official Account.

This study aims to evaluate the potential effectiveness of the mHealth supportive care intervention, with respect to supporting parents of children with ALL on emotional distress, social support, care burden, uncertainty, quality of life, and knowledge. In addition, this study examined parents’ experience of receiving this mHealth supportive care intervention.

## Methods

### Participants

We recruited 101 parents of children with ALL from Shanghai Children’s Medical Center and Soochow University Affiliated Children’s Hospital to participate in this study. The first 50 parents were assigned to the observation group, followed by 51 parents to the intervention group 3 months later. The rationale for the assignment method is provided in the “Study Design” section.

As the CA app has its target users, the eligibility criteria for participants included the following: parents were the main caregiver of a child diagnosed with ALL at the age <15 years; the child had been diagnosed ALL within the past 30 days at the time when the parent was enrolled in the study; parents had the education of high school or higher; parents were able to communicate fluently in Mandarin Chinese; patient’s average monthly family income was ≥2000 Chinese yuan (equivalent to US $300); parents were willing to initiate and continue ALL treatment for the child; and parents had an Android smartphone and a frequently used WeChat account. The exclusion criteria included the following: the child had other severe diseases or chronic diseases and the child is in a critical condition.

The study received ethical review approval from the Second Military Medical University, Shanghai Children’s Medical Center, and Soochow University, China. Participants attended an information session and were informed of the aim of this study and participant’s rights. All participants provided written informed consent prior to their participation in this study.

### Study Design

A quasi-experiment (nonrandomized experiment) pre- and postdesign was used to evaluate the potential effectiveness of the mHealth supportive care intervention. The study was conducted from June 2015 to January 2016. To prevent parents in the observation group from getting the CA app and WeChat Official Account through interacting with parents in the intervention group and consequently resulting in contamination in data collection, a 3-month data collection of the observation group was carried out first, followed by the intervention trial. Parents in the observation group received the standard health education in the hospital and were observed for 3 months. The standard health education in hospital included regular health education sessions, which were available to all caregivers of children, health education fliers or booklets, and one-on-one education by nurses. The standard health education neither included any information about the CA app, WeChat Official Account, nor the mHealth intervention. Parents in the intervention group received the mHealth supportive care intervention, in addition to the standard health education. The intervention period lasted 3 months.

### Intervention of the Mobile Health Supportive Care

Study researchers helped parents in the intervention group install the CA app and trained parents using the app. In addition, parents subscribed the WeChat Official Account used for this study. Parents used the app freely during the intervention period and also received health education papers sent through the WeChat Official Account. Furthermore, parents could communicate with our care management team with questions about caring for their children.

The care management team included 4 members—1 software engineer, 1 clinical nurse, and 2 nursing researchers. The team performed following functions: maintaining the CA app to ensure that the app functioned well without software failure; selecting and distributing weekly supportive care health education papers in 3-5 topics, based on parents’ needs and clinical judgments, to parents through the WeChat Official Account, with one paper being an example of successful ALL treatment; and answering parents’ questions through the WeChat Official Account.

### Outcome Measures

We applied 7 measures to assess the effectiveness of the mHealth supportive care. The 7 measures are defined below.

#### Anxiety

Zung’s Self-Rating Anxiety Scale (SAS) was used to measure anxiety. The SAS is a 20-item, 4-point scale ranging from never (1) to often (4) [19]. The total score of 1.25 is the final standard score. A higher score indicates a severer anxiety symptom. The Chinese version of the SAS is widely used in China, and its reliability has been established (Cronbach alpha=.85) [19,20].

#### Depressive Disorder

Zung’s Self-Rating Depression Scale (SDS) was used to measure depression. The SDS is a 20-item, 4-point scale ranging from never (1) to often (4) [21]. The total score of 1.25 is the final standard score. A higher score indicates severer depressive symptoms. The Chinese version of SDS is with good reliability and widely used among Chinese populations (Cronbach alpha=.86) [19].

#### Perceived Social Support

The Perceived Social Support Scale (PSSS) was used to measure perceived social support [22]. The PSSS is a 12-item, 7-point scale ranging from never (1) to every day (7). A lower score indicates poorer social support. The Chinese version of the PSSS has demonstrated good reliability in prior studies (Cronbach alpha=.88) [23].

#### Burden of Care

The Zarit Burden Inventory (ZBI) was used to measure parents’ burden of care [24]. The ZBI is a 22-item, 5-point scale ranging from never (0) to always (4). A higher score indicates higher care burden. The Chinese version of the ZBI has demonstrated good reliability (Cronbach alpha=.87) [24], and was used in caregivers of Chinese adult patients with ALL [25].

#### Parents’ Perception of Uncertainty in Illness

The Parents’ Perception of Uncertainty Scale (PPUS) was used to measure parents’ uncertainty in illness [26]. The PPUS is a 28-item, 5-point scale ranging from strongly disagree (1) to strongly agree (5). A higher score indicates a stronger perception of uncertainty. The Chinese version of the PPUS is with good reliability (Cronbach alpha=.84) [26].

#### Quality of Life

The Medical Outcomes Study 36-item Short Form (SF-36) was used to measure the parents’ quality of life [27]. The SF-36 comprises 36 items summarized into 8 dimensions—physical functioning, social functioning, pain, mental health, vitality, general health, role limitation owing to physical problems, role limitation owing to emotional problems, and a single-item subscale on health transition. The Chinese version of the SF-36 is widely used with Cronbach alpha coefficients ranging from.72 to.88, except.39 for the social functioning scale and.66 for the vitality scale [27]. In this study, we used the 8 validated subscales of the SF-36.

#### Parents’ Existing Knowledge and Knowledge Needs

Parents’ existing knowledge of ALL and care and their need for related knowledge were measured by the Knowledge Questionnaire. The questionnaire was designed by the authors and had 2 parts—existing knowledge and knowledge needs. The existing knowledge and knowledge needs subscales have the same 50 items. For the existing knowledge part, it is a 5-point scale ranging from “do not know at all” (1) to “know very well” (5). A lower score indicates less existing knowledge. For the knowledge needs part, it is a 5-point scale ranging from “do not want to know at all” (1) to “want to know very much” (5). A higher score indicates higher knowledge needs.

### Data Collection

#### Observation Group

Data were collected at 2 time-points—at the beginning (preobservation data collection) and the end (postobservation data collection) of the 3-month study period. In the preobservation data collection, 2 sets of the data were collected as follows: (1) parents’ and their child’s sociodemographic characteristics pertinent to the study, collected using an in-house developed questionnaire and (2) outcome data as defined in the preceding “Outcome Measures” section. In the postobservation data collection, only the outcome data were collected in the same way as in the preobservation data collection. In both data collections, participants answered the questionnaires according to their experience in the previous week. No exit interviews were conducted among participants in the observation group because interviews were held to collect parents’ experience of receiving the intervention, and parents in the observation group did not receive any intervention.

#### Intervention Group

Similar to the observation group, the following additional data collection, semistructured one-on-one and face-to-face interviews with active participants of the intervention group, were conducted in the postintervention data collection. The purpose of the interviews was to understand parents’ experience of and attitude toward receiving this intervention and their willingness of receiving this intervention in the future. Moreover, parents were asked to talk about their support needs and if they had unmet needs still to be addressed. The interviews were conducted one at a time until data saturation was achieved [28]. In total, 11 parents were interviewed. This was a triangulation mixed-methods design. The qualitative data we chose to collect included important information that could help to demonstrate the effectiveness of this study and explain the quantitative results. Researchers obtained parents’ true feelings of the intervention, why parents felt they benefited from the intervention, and more suggestion to improve further intervention after this potential effectiveness study.

### Data Analysis

#### Quantitative Data

Data collected from 43 participants in the observation group and 49 participants in the intervention group were analyzed. All data were entered into a centralized database and analyzed with IBM SPSS 21.0 statistical software. Descriptive data were presented as mean (SD) for continuous variables, and as absolute and relative frequencies for categorical variables. The comparison of the baseline sociodemographic characteristics in the 2 groups was made using the independent- sample *t* test or one-way analysis of variance. The difference in outcomes between the 2 groups was examined using the independent-sample *t* test or paired-sample *t* test. The level of significance was set at alpha=.05.

#### Qualitative Data

The audiorecording of the qualitative data was transcribed and analyzed by 2 researchers independently within 24 hours after the interview. A thematic framework of content analysis was applied to analyze the information [28]. In addition, a stepwise approach was adopted for the content analysis. First, the transcribed data were read several times by the researchers to find the theme of the whole. Second, the segmentation of information was done to organize the segments and subsegments of information. Third, significant information related to research questions was extracted. Finally, data were coded and grouped into categories and abstracted into subthemes and the main theme.

## Results

### Overview of Participants at Each Stage of the Evaluation Progression

**Figure 3:**
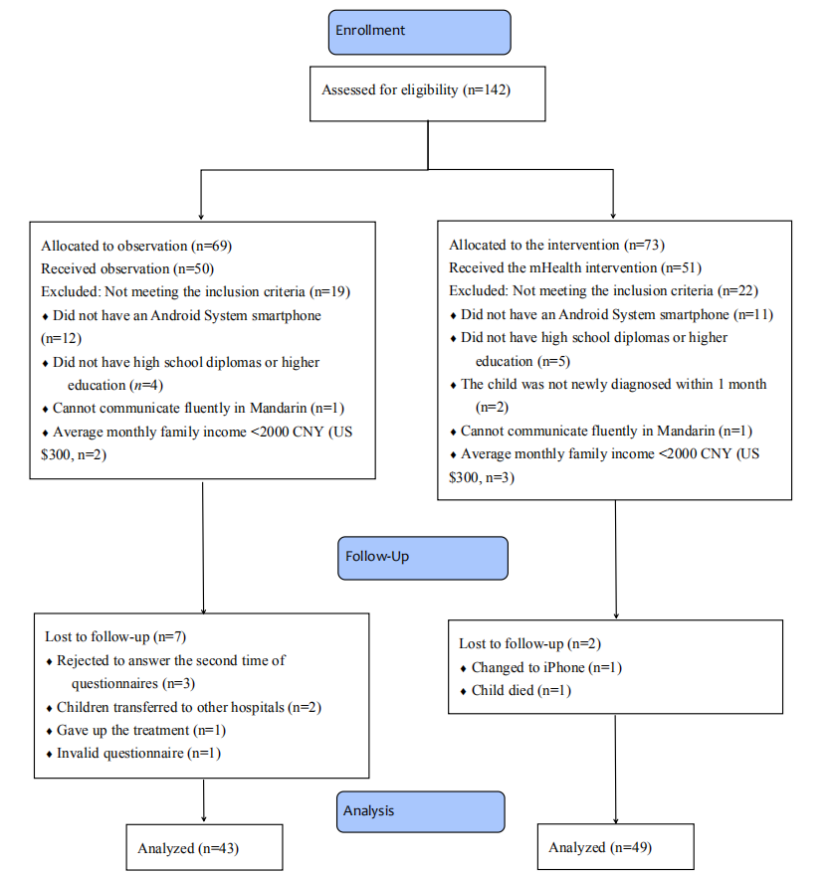
The progression of participants at each stage of the evaluation. CNY: Chinese Yuan.

A total of 50 parents in the observation group and 51 parents in the intervention group participated in this study. In the observation group, 86% (43/50) parents completed the study. Among 7 parents in the observation group who did not complete the study, 3 parents rejected to complete the data collection in the postobservation phase, 2 children transferred to other hospitals, 1 child’s parents gave up their child’s treatment, and 1 parent’s data were invalid for the postobservation phase questionnaire was not finished by the only parent who was being surveyed and observed. In the intervention group, 96% (49/51) parents completed the study. Two parents did not complete the study as 1 parent stopped using Android smartphone on which the CA app only works, and the other parent’s child died during the study period. Figure 3 displays the progression of participants at each stage of the evaluation.

### Sociodemographic Characteristics

Multimedia Appendix 1 summarizes participants’ and their children’s baseline sociodemographic characteristics. No significant differences were observed between the intervention and observation groups (*P*>.05).

### Baseline Outcome Questionnaire Results

Table 1 shows the baseline outcome questionnaire results. The data were collected during the preintervention data collection. There were no significant differences in questionnaire results at the baseline between the intervention and observation groups (*P*>.05).

**Table 1:** The comparison of participants’ baseline questionnaire results.

Name of the scales		Intervention group (n=49), mean (SD)	Observation group (n=43), mean (SD)	*t* value	*P* value
Perceived Social Support Scale		66.7 (10.0)	65.2 (11.5)	0.644	.52
Self-Rating Anxiety Scale		49.8 (10.7)	49.5 (10.8)	0.155	.88
Self-Rating Depression Scale		56.9 (12.1)	55.9 (13.7)	0.377	.71
Zarit Burden Inventory		37.6 (10.4)	37.2 (11.1)	0.180	.86
Parents’ Perception of Uncertainty Scale		114.4 (7.1)	113.7 (6.8)	0.507	.61
**Medical Outcomes Study 36-item Short Form**					
	Physical functioning	85.6 (18.2)	86.2 (19.6)	−0.140	.89
	Social functioning	69.6 (23.4)	70.6 (23.0)	−0.206	.84
	Pain	77.6 (17.8)	77.5 (20.5)	0.020	.98
	Mental health	58.1 (18.3)	58.4 (22.9)	−0.069	.95
	Vitality	58.5 (19.5)	58.4 (20.1)	0.024	.98
	General health	64.2 (20.6)	66.0 (19.3)	−0.428	.67
	Role limitation owing to physical problems	39.8 (46.5)	43.6 (47.3)	−0.389	.70
	Role limitation owing to emotional problems	31.9 (42.4)	29.4 (41.9)	0.285	.78
**Knowledge questionnaire**					
	Existing knowledge	23.3 (5.0)	23.6 (7.3)	−0.259	.80
	Knowledge needs	98.9 (4.1)	99.5 (3.1)	−0.807	.42

### The Effectiveness of the Mobile Health Supportive Care Intervention

#### Comparison of Outcome Questionnaires Results’ D Value (Postintervention-Preintervention) Between the Intervention and Observation Groups

Multimedia Appendix 2 shows participants’ outcome questionnaires results’ *D* values (postintervention- preintervention) in the intervention and observation groups. Results showed that the mHealth supportive care intervention significantly reduced parents’ anxiety (*P*=.03), reduced uncertainty in illness (*P*=.01), improved parents’ social function (*P*=.01), increased parents’ knowledge of ALL and care (*P*<.001), and decreased parents’ need of knowledge (*P*<.001).

#### Comparison of Preintervention and Postintervention Questionnaires Results in the Intervention Group

Multimedia Appendix 2 presents preintervention and outcome questionnaire results in the intervention group. The findings showed that after the 3-month intervention, parents’ anxiety (*P*<.001), depression (*P*=.01), uncertainty in illness (*P*<.001), and knowledge needs significantly decreased(*P*<.001), and the knowledge of ALL and care parents had significantly increased (*P*<.001).

#### Comparison of Preintervention and Postintervention Questionnaires Results in the Observation Group

Multimedia Appendix 2 shows preintervention and postintervention questionnaire results in the observation group. The results showed that over the course of 3-month observation, parents’ care burden significantly increased (*P*=.01), and the knowledge of ALL and care significantly increased (*P*<.001), whereas uncertainty in illness significantly decreased (*P*<.001).

### Qualitative Results

Eleven parents in the intervention group were interviewed regarding their experience of, attitude toward, and willingness to participate in the intervention. While 4 parents were fathers, 7 were mothers of children with ALL. Parents aged 22-35 years with the education of high school or above. Their family income ranged from 2000 to 8000 Chinese yuan a month. Their children aged 1-7 years, and 6 of them were boys. Multimedia Appendix 3 shows the detailed qualitative data by category. The qualitative results indicated that the overall experience of receiving this mHealth supportive care intervention was positive. Parents agreed that this intervention provided them useful information of ALL and care for their children. They were benefited from receiving this intervention but still had unmet supportive care.

## Discussion

### Principal Findings

This study indicated the potential effectiveness of the mHealth intervention in supporting parents of children with ALL. Results found that the intervention reduced parents’ anxiety and uncertainty in illness, improved parents’ social function, increased parents’ knowledge of ALL and care, and decreased their need for knowledge. Parents were satisfied with the intervention and their role in the caregiving process. Moreover, this study is informative for other future studies on providing mHealth supportive care for parents of children with cancer.

Parents of children with ALL are engaged with a variety of care activities because of the special characteristics of ALL and children’s limited cognitive ability. Studies have indicated parents’ supportive care needs and poor health status [7]. To provide parents with accessible and effective supportive care is an urgent need. Interventions provided through smartphone apps have been proved to be feasible and effective in the health management of patients with chronic diseases [12]. However, few studies have focused on providing supportive care intervention to caregivers of patients with cancer, particularly children with cancer. Thus, this study aimed at providing supportive care to parents of children with ALL using mHealth. This is an innovative approach to meet parents’ needs. Our research team took the lead in developing a mHealth smartphone app to support parents when the methodology and clinical application of mHealth interventions were at the very beginning stage in China.

The intervention was developed with rigorous methodology. A qualitative study with parents of children with ALL was initially conducted to gain an in-depth understanding of the characteristics and supportive care needs of parents [29]. The CA app was then developed according to a user-centered development process with a multidisciplinary group including software engineers and researchers [17]. A scoping review of the usability evaluation of mobile apps for cancer was conducted, which guided the app’s usability evaluation. Parents, physicians, and nurses participated in the usability evaluation. Their use experience data were collected and analyzed [18]. Guided by the supportive care framework and based on parents’ needs, preference, and the usability evaluation results, this mHealth supportive care intervention was developed. Then, an optimization discussion with professional health care providers was conducted to refine the intervention. This systematic process promoted the effectiveness of the intervention.

We prototyped a multicomponent mHealth intervention program to support parents. Based on the suggestions of health care providers, we had the CA app as the main intervention tool. “WeChat,” the most popular chat tool in China, was added to the intervention to enable communication between parents and the care management team and provide the information that is not available in the app. Two intervention components work collaborated to provide parents with accessible and convenient supportive care and meet parents’ constantly changing needs. The multidisciplinary care management team, including nurses, researchers, and software engineers, facilitates communication between parents and professional health care providers with high efficiency.

In the outcome measurement, we included both quantitative and qualitative outcomes. The quantitative results demonstrated the potential effectiveness of this mHealth supportive care intervention in improving parents’ psychological health status, quality of life, care satisfaction and confidence, knowledge of ALL, and care for children. The effectiveness is in accordance with traditional interventions’ results [8-10]. In this study, parents accepted and were willing to go on using this app and the WeChat Official Account. In the interviews, parents mentioned several times about the ease of access to scientific knowledge about the disease and care for their children, which improved their care confidence and communication efficiency with physicians and nurses. The easy access of information increased parents’ existing knowledge and decreased their knowledge needs, which matched with the quantitative data.

In addition, accessible information and response from the care management team helped to reduce parents’ anxiety and uncertainty of illness. Parents mentioned that there were professional health care providers to answer their personalized questions, which made them feel safe and less anxious. Parents felt they were more confident in handling some problems they encounter during the caring process. However, depression is a more serious and complicated psychological symptom. The intervention group parents’ depression symptom decreased significantly (*P*=.08), whereas there was no significant decrease in the observation group nor the *D* value between the 2 groups (*P*=.08, *P*=.83). These results indicated that more professional psychological intervention need to be added in our mHealth intervention and further psychological effectiveness of the intervention need to be evaluated.

With the progress of children’s disease and treatment, parents need to take on more care responsibilities and also have the heavier economic burden, which would increase parents’ overall care burden. The observation group parents’ care burden increased significantly (*P*=.09), whereas there was no significant increase in the intervention group and no significant difference between the *D* value in the 2 groups (*P*=.62, *P*=.08). Parents in the intervention group got more guide and direction from the CA app, WeChat Official Account, and professional health care providers, which might lead to better care for children, fewer complications, less treatment fee, or less increased care burden. The reason for a change in the care burden need to be explored in further study.

This intervention did not provide effective social support to parents, as the change of parents’ perceived social support did not increase significantly (*P*=.94). However, the intervention group parents’ social function improved compared with the observation group (*P*=.01). However, the intervention was still far from improving parents’ quality of life because the other dimensions of quality of life did not change significantly: physical functioning (*P*=.56), pain (*P*=.66), mental health (*P*=.95), vitality (*P*=.47), general health (*P*=.86), role limitation owing to physical problems (*P*=.64), and role limitation owing to emotional problems (*P*=.32). These results indicated that family and friends’ support needs to be improved in further study and have more family members participating in different aspects of caring for the child may be helpful for parents’ physical and psychological status. Furthermore, the quality of life is a long-term indicator, which may not change immediately with the intervention, so further effectiveness evaluation is necessary.

Moreover, with this intervention, parents still expressed needs that were to be met, such as the need of an app to run on iOS phones, long-term support, and the worry about their children’s disease progression and relapse. To a certain degree, these qualitative results explain why parents need more disease-related knowledge, though the need decreased in comparison with the preintervention period. These qualitative results help explain causes for the quantitative results and provide us with information about the plausible relationship between the variables. This mixed-methods design provides us with the advantage of comprehensively examining the effectiveness of the intervention.

This intervention met parents’ needs and improved the efficiency of communication between health care providers and parents. Information on patient care provided in this intervention has the potential effectiveness in helping reduce health care providers’ educational burden and provided an approach to standardizing health education. Basic disease-related information decreased parents’ frequency of asking health care providers common questions. However, parents always hope there could be someone reliable that would respond to them when they occurred with some new personalized problem. We got this need from our former study [17,29], and this is why we set the care management team with clinical nurse and nursing researchers. Further evaluation of the intervention’s influence on professional health care providers will be conducted. Moreover, effective and efficient communication between professional health care providers and parents is essential to build a harmonious patient-provider relationship, which highlights the promotional value of further clinical application of this mHealth supportive care intervention.

### Limitations and Future Directions

The CA app currently runs only on an Android smartphone. Many parents use iOS phones. A CA app running on iOS will be developed in the near future. This study was a nonrandomized controlled trail. In the future, we will address the issue of cross-group contamination and conduct a randomized controlled study to validate the intervention effectiveness. The CA app had its target users, which led to this mHealth supportive intervention being not suitable for every parent; only parents with basic education background can fully understand the text content in the CA app and WeChat Official Account. This study was conducted for 3 months with a sample size of about 50 for each group. The effect of intervention over a longer course of care continuum on all caregivers is yet to be assessed. In future studies, we will increase the sample size by recruiting main caregivers, including parents and other people, and extend the observation period to the entire treatment course.

### Conclusions

This mHealth supportive care intervention showed its potential effectiveness in reducing parents’ anxiety and uncertainty in illness, improving parents’ social function, increasing parents’ knowledge of ALL and care for children, and decreasing parents’ need of information. Parents are satisfied with this intervention and willing to continue receiving the intervention. This study presents informative methodologies of assessing the effectiveness of providing supportive care to parents of children with cancer through a mHealth intervention.

## Appendix

Multimedia Appendix 1 Participants’ baseline sociodemographic characteristics stratified by the study group.

Multimedia Appendix 2 Comparison of outcomes’ changes in the intervention group (n=49), in the observation group (n=43), and D value between the 2 groups.

Multimedia Appendix 3 The qualitative results from the interviews in the intervention group (n=11).

## Abbreviations

ALL: acute lymphoblastic leukemia
CA: Care Assistant
mHealth: mobile health
PPUS: Parents’ Perception of Uncertainty Scale
PSSS: Perceived Social Support Scale
SAS: Self-Rating Anxiety Scale
SDS: Self-Rating Depression Scale
SF-36: Medical Outcomes Study 36-item Short Form
ZBI: Zarit Burden Inventory
