# Multimedia Appendix Correction: mHealth Supportive Care Intervention for Parents of Children With Acute Lymphoblastic Leukemia: Quasi-Experimental Pre- and Postdesign Study

**DOI:** 10.2196/13159

**Published:** 2019-02-20

**Authors:** Jingting Wang, Doris Howell, Nanping Shen, Zhaohui Geng, Fulei Wu, Min Shen, Xiaoyan Zhang, Anwei Xie, Lin Wang, Changrong Yuan

**Affiliations:** 1 School of Nursing Second Military Medical University Shanghai China; 2 Department of Supportive Care Princess Margaret Cancer Center Toronto, ON Canada; 3 Faculty of Nursing University of Toronto Toronto, ON Canada; 4 Department of Nursing Shanghai Children’s Medical Center Shanghai China; 5 Department of Hematology Children’s Hospital of Soochow University Suzhou China; 6 Department of Hematology and Oncology Shanghai Children’s Medical Center Shanghai China; 7 Department of Infectious Disease Children’s Hospital of Soochow University Suzhou China; 8 United States Census Bureau Washington, DC United States; 9 School of Nursing Fudan University Shanghai China

The authors of mHealth Supportive Care Intervention for Parents of Children With Acute Lymphoblastic Leukemia: Quasi-Experimental Pre- and Postdesign Study (JMIR Mhealth Uhealth 2018;6(11):e195) noticed problems with all three Multimedia Appendices which were published alongside the article as supplementary files. The link for Multimedia Appendix 1 was broken and would not allow the file to be downloaded, while the contents of Multimedia Appendices 2 and 3 were incorrect. All three Multimedia Appendices have been replaced with the appropriate files and the links have been confirmed as functional.

The correction will appear in the online version of the paper on the JMIR website on February 20, 2019, together with the publication of this correction notice. Because this was made after submission to PubMed, PubMed Central, and other full-text repositories, the corrected article also has been resubmitted to those repositories.

## Appendix

Multimedia Appendix 1 Participants’ baseline sociodemographic characteristics stratified by the study group.

Multimedia Appendix 2 Comparison of outcomes’ changes in the intervention group (n=49), in the observation group (n=43), and D value between the 2 groups.

Multimedia Appendix 3 The qualitative results from the interviews in the intervention group (n=11).

