# Supplementary material for: mHealth Supportive Care Intervention for Parents of Children With Acute Lymphoblastic Leukemia: Quasi-Experimental Pre- and Postdesign Study
Source: JMIR Mhealth Uhealth. 2018 Nov 19;6(11):e195. doi: 10.2196/mhealth.9981 (PMC6301810; doi:10.2196/mhealth.9981)
Supplement: Multimedia Appendix 1 [file mhealth_v6i11e195_app1.pdf]

Multimedia Appendix 1

Participants' baseline sociodemographic characteristics stratified by the study group.

Participant characteristics		Intervention group (n=49) n (%)	Observation group (n=43) n (%)	*t* or *F* value	*P* value
**Gender**				t_{90}= 0.998	.32
	Male	16 (33)	10 (23)		
	Female	33 (67)	33 (77)		
**Age**				F_{90}= 0.683	.71
	≤30 y	16 (33)	16 (37)		
	31-40 y	29 (59)	22 (51)		
	>40 y	4 (8)	5 (12)		
**Nationality**				t_{90}= 0.887	.35
	Han Nationality	48 (98)	43 (100)		
	Ethnic Minority	1 (2)	0 (0)		
**Education**				F_{90}= 2.958	.23
	High School	27 (55)	22 (51)		
	Junior College	8 (16)	13 (30)		
	Bachelor degree or above	14 (29)	8 (19)		
**Marital status**				F_{90}=5.292	.71
	Single	1 (2)	2 (5)		
	Married	47 (96)	35 (81)		

	Divorced or separated	1 (2)	6 (14)		
**Chronic disease**				t_{90}= 1.024	.31
	Yes	2 (4)	4 (9)		
	No	47 (96)	39 (91)		
**Religion**				F_{90}= 5.824	.12
	None	33 (67)	33 (77)		
	Buddhism	13 (27)	8 (18)		
	Christianism	3 (6)	0 (0.0)		
	Others	0 (0)	2 (5)		
**Original living place**				t_{90}= 0.827	.36
	City	24 (49)	17 (40)		
	Country	25 (51)	26 (60)		
**Whether travel to other city to have treatment**				t_{90}= 0.011	.92
	Yes	36 (73)	32 (74)		
	No	13 (27)	11 (26)		
**Employment status**				F_{90}= 7.159	.07
	Full time	25 (51)	14 (33)		
	Part time	12 (25)	17 (39)		
	Not work	11 (22)	7 (16)		
	Others	1 (2)	5 (12)		
**Profession**				F_{90}= 2.972	.56

	General worker	10 (20)	12 (28)		
	Farmer	9 (19)	10 (23)		
	Administrative staff	2 (4)	0 (0.0)		
	Teachers	2 (4)	1 (2)		
	Others	26 (53)	20 (47)		
**Average monthly family income**				F_{90}= 4.304	.12
	2000-5000 CNY[a]	28 (57)	33 (77)		
	5000-8000 CNY	11 (23)	4 (9)		
	>8000 CNY	10 (20)	6 (14)		
**Payment for treatment**				F_{90}= 0.480	.93
	Self-pay	27 (55)	24 (56)		
	Medical health insurance	13 (27)	13 (30)		
	Rural cooperative health care insurance	8 (16)	5 (12)		
	Others	1 (2)	1 (2)		
**Daily care time**				F_{90}= 0.310	.96
	<6 h	2 (4)	1 (2)		
	6-12 h	10 (21)	10 (23)		
	12-18 h	6 (12)	5 (12)		
	18-24 h	31 (63)	27 (63)		
**Number of cocaregivers**				F_{90}= 6.619	.09
	0	0 (0)	2 (5)		

	1	31 (63)	34 (79)		
	2	10 (21)	4 (9)		
	3 or more	8 (16)	3 (7)		
**Gender of child**				t_{90}= 0.385	.54
	Boy	30 (61)	29 (67)		
	Girl	19 (39)	14 (33)		
**Age of child**				F_{90}=1.339	.86
	<1	4 (8)	3 (7)		
	1-3	9 (19)	6 (14)		
	3-7	24 (49)	19 (4)		
	7-12	7 (14)	8 (19)		
	12-18	5 (10)	7 (16)		
**Education of child**				F_{90}=2.494	.29
	Below primary school	36 (74)	25 (58)		
	1-2 grades of primary school	5 (10)	6 (14)		
	3 grades of primary school or above	8 (16)	12 (28)		
