# Supplementary material for: mHealth Supportive Care Intervention for Parents of Children With Acute Lymphoblastic Leukemia: Quasi-Experimental Pre- and Postdesign Study
Source: JMIR Mhealth Uhealth. 2018 Nov 19;6(11):e195. doi: 10.2196/mhealth.9981 (PMC6301810; doi:10.2196/mhealth.9981)
Supplement: Multimedia Appendix 2 [file mhealth_v6i11e195_app2.pdf]

Multimedia Appendix 2

Comparison of outcomes' changes in the intervention group (n=49), in the observation group (n=43), and D value between the 2 groups.

	Intervention Group (n=49) Mean (SD)					Observation Group (n=43) Mean (SD)					*t*	*P* value
	Pre	Post	*D* value (Post-Pre)	*t*	*P* value	Pre	Post	*D* value (Post-Pre)	*t*	*P* value		
Perceived Social Support Scale	66.7 (10.0)	66.6 (11.7)	–0.1 (12.7)	0.056	.96	65.2 (11.5)	64.9 (10.5)	–0.3 (17.2)	0.124	0.90	0.071	.94
Self-Rating Anxiety Scale	49.8 (10.7)	42.8 (10.2)	–7.0 (13.1)	3.745	.00[a]	49.5 (10.8)	49.1 (12.2)	–0.4 (15.8)	0.157	0.88	–2.200	.03[a]
Self-Rating Depression Scale	56.9 (12.1)	50.8 (12.0)	–6.1 (15.0)	2.830	.007[a]	55.9 (13.7)	50.6 (12.9)	–5.3 (19.0)	1.812	0.08	–0.221	.83
Zarit Burden Inventory	37.6 (10.4)	38.8 (12.6)	1.2 (16.2)	–0.502	.62	37.2 (11.1)	44.5 (14.2)	7.3 (17.1)	–2.794	0.008[a]	–1.764	.08
Parents' Perception of Uncertainty Scale	114.4 (7.1)	89.4 (10.4)	–25.0 (8.2)	21.314	.00[a]	113.7 (6.8)	93.9 (7.7)	–19.8 (10.1)	12.813	0.00[a]	–2.761	.007[a]
**Medical Outcomes Study 36-item Short Form**												
Physical functioning	85.6 (18.2)	86.7 (13.8)	1.1 (23.5)	–0.335	.74	86.2 (19.6)	84.3 (18.7)	–1.9 (25.7)	0.475	0.64	0.582	.56

	Social functioning	69.6 (23.4)	78.6 (19.3)	9.0 (32.8)	–1.907	.06	70.6 (23.0)	63.1 (21.6)	–7.5 (30.3)	1.637	0.11	2.494	.01[a]
	Pain	77.6 (17.8)	81.6 (15.8)	4.0 (23.4)	–1.199	.24	77.5 (20.5)	79.1 (15.8)	1.6 (28.2)	–0.373	0.71	0.447	.66
	Mental health	58.1 (18.3)	64.0 (18.9)	5.9 (24.8)	–1.657	.10	58.4 (22.9)	64.7 (18.8)	6.2 (24.6)	–1.658	0.11	–0.069	.95
	Vitality	58.5 (19.5)	63.5 (21.6)	5.0 (26.4)	–1.328	.19	58.4 (20.1)	59.4 (24.4)	1.0 (25.9)	–0.265	0.79	0.724	.47
	General health	64.2 (20.6)	66.7 (20.2)	2.5 (21.9)	–0.796	.43	66.0 (19.3)	67.5 (21.7)	1.5 (28.6)	–0.346	0.73	0.182	.86
	Role limitation owing to physical problems	39.8 (46.5)	31.1 (41.9)	–8.7 (56.7)	1.070	.29	43.6 (47.3)	40.7 (43.0)	–2.9 (62.0)	0.307	0.76	–0.466	.64
	Role limitation owing to emotional problems	31.9 (42.4)	47.6 (43.5)	15.7 (63.1)	–1.735	.09	29.4 (41.9)	31.8 (39.8)	2.4 (63.9)	–0.238	0.81	1.003	.32
**Knowledge Questionnaire**													
	Existing knowledge	23.3 (5.0)	51.7 (12.2)	28.4 (12.4)	–15.997	.00[a]	23.6 (7.3)	40.8 (9.8)	17.2 (11.9)	−9.448	.00[a]	4.407	.00[a]
	Knowledge needs	98.9 (4.1)	89.0 (11.2)	–9.9 (11.6)	5.948	.00[a]	99.5 (3.1)	97.6 (5.5)	−1.9 (6.4)	1.981	0.05	−4.112	.00[a]

[a]Data were statistically significant at the .05 level (2-tailed).
