# Supplementary material for: mHealth Supportive Care Intervention for Parents of Children With Acute Lymphoblastic Leukemia: Quasi-Experimental Pre- and Postdesign Study
Source: JMIR Mhealth Uhealth. 2018 Nov 19;6(11):e195. doi: 10.2196/mhealth.9981 (PMC6301810; doi:10.2196/mhealth.9981)
Supplement: Multimedia Appendix 3 [file mhealth_v6i11e195_app3.pdf]

Multimedia Appendix 3

The qualitative results from the interviews in the intervention group (n=11)

**1. The overall experience of receiving this mHealth supportive care intervention**		
	1.1 Helpful, necessary and reliable intervention	“This study is so helpful! When we knew my child had this disease, I was shocked. I have no idea of what to do. But you app provided me a lot of information, you answered my questions. Thank you so much for what you have done. ” (P1)
		“It is so necessary to provide us, parents of ALL children such information and support. There is nobody in our family study medical or have any related experience. We do not know anything of ALL. Thank you for all your help.” (P5)
		“I like this project. The app and WeChat(Official Account) are so helpful for me.” (P8)
		“I don’t think your WeChat information bother us. The information is helpful. Once you sent (the information article), I read it.” (P4)
		“You really provided so much help to me. The app and the WeChat (Official Account) information are very useful.” (P3)
**2.Perceived benefits from the intervention**		
	2.1 Easy access to disease-related information	“The knowledge in this app is comprehensive, very helpful for me. I read it when I am free.” (P10)
		“Many problems come out when I am taking care of my child. When I came across with any problem, I tried to find the answer in the app. If there is answer in the app, I don’t have to bother the doctors or nurses. That’s very convenient.” (P4)
		“I knew nothing of this disease at the beginning. The hospital gave us health education classes. But I can’t remember all the information they provided. When I have this app and your WeChat Official Account, I remember the knowledge little by little.” (P7)
		“When I search for the Internet, there is so much information. I don’t know which one is correct, which one is wrong. Your information (in the app and WeChat Official Account) is better, it is the same as the health education in the hospital. Also, it is very convenient and easy to get. ” (P6)
		“It is easier to use your app than search the Internet, and the knowledge is correct and credible.” (P9)
	2.2 Better understanding of ALL and care knowledge	“I have no idea of the disease when she (the child) was diagnosed. Now I know more information and know how to handle some situations. Your app is really helpful.” (P8)
		“After using this app, I have more knowledge of ALL.” (P10)
		“Compare to the very beginning, when (the child was) firstly diagnosed, I really know much more knowledge.” (P7)
		“I just finished your Knowledge Questionnaire, one question by one question, I find that I have much more knowledge.” (P3)
	2.3 Improvement of confidence	“I am more confident than before, I can solve some problems by myself.” (P4)
		“I know what medicine my child is using, and I know what kind of side effects he may have, even what I can do for him.” (P6)

	in care	"I like the WeChat Official Account. There is information on diet and menu. I cooked as the menu, and I am happy because sometimes my child likes it." (P7)
		"When we finished the treatment in hospital and went back home, it was very inconvenient to ask the doctors and nurses questions. Then I felt this app and the WeChat Official Account was very helpful. I asked (by the WeChat Official Account) when I met some problems, there was someone answering me. I did as they told and I felt I had more confidence." (P10)
	2.4 Decrease of anxiety and uncertainty of illness	"I got a lot of information in your app and WeChat Official Account. I know more about the disease, and I am not as anxious as the beginning." (P1)
		"As I know more information, I am not so afraid. What I can do is to do as the doctor said." (P3)
		"I know more and I am not as anxious and afraid as before." (P8)
		"I am not very afraid. Just step by step, after we finish the chemotherapy, I hope my child could get better." (P2)
	2.5 Better communicatio n with health care providers	"I have some basement knowledge of the disease, then I can understand doctors and nurses' explanation and education better" (P7)
		"I can't understand what the doctor said when (the child was) diagnosed. Now I am much better." (P6)
		"I can understand nurses' explanation." (P3)
		"When I have questions, I always try to find the answer in the app first. If I still have something not understand, I ask the doctor target questions." (P4)
**3.Unmet supportive care needs**		
	3.1 An app for iOS system	"My husband's phone is an iPhone, he can't use the app. He always uses my phone to get more information." (P8)
		"I changed my phone (changed to an iPhone) because it (the Android system phone) had some problems during your study. And I have to borrow one Android smartphone to use your app. That is not very convenient." (P9)
		"This app is very good, but can't be used on iPhone. If you could develop an app for iPhone, it would be so great." (P5)
	3.2 Long-term professional support	"Your study finished, you mean there will not be someone to answer my question? That is so pity." (P3)
		"Your study ended? If I have questions, can I still ask you for help?" (P6)
		"I hope there could be someone answering my question continually, I have some knowledge, but I want more." (P5)
		"I sincerely hope there is someone always at the backstage and answer our questions" (P10)
	3.3 Anxiety about disease progression, especially for relapse	"Now we can only do as the doctor said, but I am very afraid that my child get relapse." (P11)
		"See the poor boy next to us (relapse case), so poor...... Do you know whether there is any sign for relapse?" (P2)
		"Actually, we have heavy economy burden, we borrow money for the treatment. If my boy relapses, I really don't know what we could do." (P4)
